# On drafting a new Mental Health Act

**DOI:** 10.4103/0019-5545.58888

**Published:** 2010

**Authors:** James T. Antony

**Affiliations:** Department of Psychiatry, Jubilee Mission Medical College Hospital, Trissur - 5, India

## ON DRAFTING A NEW MENTAL HEALTH ACT

Since some time the Indian Psychiatric Society has decided to go for a new Mental Health Act.[1,2] Let us hope that the collective efforts of the membership of the Society would yield results that would, in the long run, be for the enduring good of mentally ill, in the country! Meanwhile, we must be conscious of the fact that in early 1990s, when the Mental Health Act-1987 arrived, our reckoning was that the new ‘Magna Carta’ would usher in an era of dignified and humane care for all those who are mentally ill.

The euphoria of that time was short-lived. Soon, many psychiatrists from all over India perceived the new Act as a huge let-down![2–5] Why such a total turnaround took place, within a short period? That too, even before most state governments had put in place ‘Mental Health Rules’, for their respective jurisdictions? These are points that need to be carefully analysed. After all, ‘the legislative policy’ of the 1987 Act, as highlighted in its ‘Statement of Objects and Reasons’, is on the whole, quite praiseworthy.

Just two words in the Act, namely ‘Licensing’ and ‘Inspectors’ are the ones that are totally unacceptable to many a critic! Of course, the word ‘licensing’ has really earned notoriety in this whole country, for many decades, probably ever since independence! Till recently, we had wide currency for phrases like ‘license-permit-raj’! And as for ‘inspector’, all statutory inspectors in this country have the odor of ‘gutter inspector’, about whom the Mahatma himself had commented about, long ago!

But despite all these, a civilized society cannot do away completely, with regulatory mechanisms, in vital sectors. It is true that in a society steeped in corruption, licensing could be a milching cow for all and sundry. But what is the remedy? Has the profession no responsibility to put in place a surveillance system that would ensure that only competent persons, committed to provide good-quality services are able to run ‘total-care’ institutions, like mental hospitals? Or is it our case that ‘as I am a qualified psychiatrist, the whole world has no business to question my honesty?’

A stand that there is no need for any kind of licensing and inspection is not something that legal experts or human right activists would accept. In these kinds of things, one has to be realistic. The mentally ill people in ‘total-care’ or ‘custodial’ hospitals are not exactly like other patients in hospitals, who mostly would be staying along with a close relative or friend. Also those patients with no mental infirmity would mostly be in a position to protect themselves against high-handedness from any quarters.

But the mentally ill are different. They would need some legal provisions, to protect their basic human rights.[3,6] This is a point that the profession has to concede. True, any licensing system needs to be rational and must have a clear purpose of realizing objectives stated in the preamble of the Act. Also, the procedure has to be simplified. The fee has to be fixed with the clear purpose of optimally regulating the system. The idea should not be, merely to bring in more money to the government kitty.

There has to be a set of ‘Rules’ that would ensure that all those noble objectives, enumerated in the ‘Statement of Objects and Reasons’, are achieved. And for this, the profession at the state level has to get actively involved to make sure that State Mental Health Rules are framed thoughtfully, taking into account local conditions also. The endeavor has to be to suggest clear-cut acceptable alternatives, rather than blindly objecting to all kinds of ‘inspections’ and ‘licensing’.

Licensing and inspection has to be limited to total care institutions, where patients stay without bystanders. Other places should only have those regulations that are applicable to all General Hospitals. Another thing is that the present flaws in the matter of appointing inspectors must be overcome. Explicit provisions are to be there, to bar non-professionals from having access to confidential medical records regarding patients, in the name of inspection.

The existing government system that is in vogue in its various departments is to have permanent inspectors, with defined geographic jurisdictions. This should not be copied, as it would breed corruption. It would be better for State Mental Health Authorities (SMHAs) to constitute panels of inspectors, one at the state-level and one each, for every district. For a particular inspection-job in hand, the SMHAs could requisite the services of two or three inspectors and give them a definite timeframe. Such a system would ensure that chances for corruption are minimized.

While putting regulatory mechanisms in place, it is important to make sure that such measures do not put the profession to avoidable hassles, which in turn may even lead to a sort of lukewarm attitude on their part. Such a thing would not be, in the long run, healthy for the whole system. After all the high morale and a positive mindset of psychiatrists is the most important ingredient of a good-quality care-delivery system for the mentally ill. Those who draft and pilot a new Act, for the welfare of one of the most disadvantaged sections in the country should not forget all these.

The laudable legislative policy envisaged in the 1987-Act of constituting SMHAs was to put in place watchdog bodies to protect basic human rights of the mentally ill everywhere, including homes and hospitals. And ordinarily, everyone would have welcomed such a surveillance system. But the manner in which all powers have been vested with Governments, by certain explicit provisions in this law is something that makes every professional quite uncomfortable.

As per Section 3 and 4 of the Act, it is the Central and the State Governments, on their own that nominates members to respective Mental Health Authorities! As per Section 4,[7] the SMHA ‘shall be subject to superintendence, direction and control of the State government’! Further, the Act has assigned the right to frame ‘Mental Health Rules’ to Central or State Governments, all by themselves! In short, the powers vested with Governments in this whole area, is really absolute and unbridled!

This approach is contrary to the aims and objectives of any good Mental Health Law. The fact that Governments are running most of the ‘custodial-care places’ or ‘total-care-hospitals’ for the mentally ill in the country is quite relevant in this regard. In Section 3 [vii] of the statement of ‘Objects and Reasons’ of the 1987-Act, it is laid down that one of the objectives is ‘to regulate the powers of the Government for establishing, licensing and controlling psychiatric hospitals and psychiatric nursing homes for mentally ill persons’.

But after proclaiming such a noble ‘Legislative policy’ in the ‘Objects and Reasons’, as one reads through the Act, nowhere else this worthwhile principle finds any mention! A close study of the chapter II of the Act as well as the rules that are framed by Central Government illustrates how the Act as a whole has gone totally in opposition to its own stated legislative policy.

The Central Mental Health Rules have prescribed that six of the authority's nine members are to be Government officers![8] And the Government Secretary/Additional Secretary is to be the chairman! In Kerala for some time, the SMHA had only two non-official members! And neither of them was a psychiatrist! Prior to that an anesthesiologist was posted as the ‘psychiatrist-member’, by the Government! The Government's contention was that as per the Act it has the right to decide which all doctors are ‘Psychiatrists’!

May be, a study of the functioning of Mental Health Authorities in other states in India would bring out similar sickening stories. All these have virtually defeated the very purpose of creating Mental Health Authorities. How could such bodies be expected to ‘regulate the powers of the Government’ in the field of mental health care? How could they secure justice and dignity to the mentally ill, a good number of who, even to this day, are incarcerating in Government Mental Hospitals?[6] As per the present Act, SMHAs have many empowering provisions to supervise and monitor the day-to-day functioning of private institutions. But nothing, just nothing is there in the Act or Rules, to regulate the functioning of government hospitals!

It is absolutely necessary that a Mental Health Authority function independently of Government, in a quasi-judicial manner. It has to be even-handed, in protecting basic human rights of patients, irrespective of whether they are in private hospitals or in government mental hospitals[5] or even in their own homes. But in the present situation, how could anyone expect a Government with unrestricted powers, be fair and impartial, in cases where Government itself is the culprit?

To set right the various defects related to Mental Health Authorities, the chapter II of the Mental Health Act has to be completely re-drafted. It is better to lay dawn in the Act itself, various details about the qualification of chairman/member, the procedure for nominating them and so on. Psychiatrists should have adequate representation in these bodies. After all they are the only group, among professionals in this field, who by their training and experience, have the required expertise in managing mental hospitals.

Legal experts as well as enlightened citizens with firsthand experience in these fields like parents or close relatives of patients could also make useful contributions. In any case this matter should not be left to the discretion of Government and its ‘Babu-log’, without any clear and concrete provisions, in the Act. Or else, any Government of our present times would pack SMHA with persons, whose only qualification is blind loyalty and obedience to their political masters who imagine that they are king-emperors!

Another discomforting feature in the Act is about psychiatric patients in general hospitals. Strict application of the provisions of the Act, as it stands now, would cause serious difficulties to this section. While all other patients could get services without any hassles, those wanting inpatient treatments by a psychiatrist in a general hospital, would have to face many hardships. Many a stringent provision of the Mental Health Act would be invoked in their case!

The same is the situation with regards to patients in some hospitals, exclusively for the mentally ill. In these institutions patients stay with their relatives, like they do in any general hospital. Though other specialties are not there, patients have the same kind of freedom, with no threat of the hospital management infringing on it. But problems arise when these places are equated with ‘total-care’ ‘psychiatric hospitals’, where, in the absence of bystanders, patients are looked after by hospital staff. This whole confusion can be cleared easily, merely by designating these places as ‘general hospital, exclusively for psychiatric patients’, rather than calling them ‘psychiatric hospitals’.

The enforcement of stringent regulations in institutions where psychiatric patients stay with their relatives is totally unnecessary. It would only be adding to the sorrow, pain and humiliation of patients and their families, especially in the background of the cruel stigma that already haunts all psychiatric illnesses! And this would be against one of the stated objectives of the Act, that ‘the mentally ill persons are to be treated like any other sick persons and the environment around them should be made as normal as possible’.

The entire issue about private general-hospital-psychiatry-units being viewed as ‘psychiatric hospitals’ can be sorted out, by making a small correction in the definition of ‘psychiatric hospitals’. In Section 2, clause [q], where psychiatric hospitals are defined, in the first sentence the reading is ‘by the Government or any other person’. But in the last sentence of the same definition the clause ‘or any other person’ is not there, after ‘by the Government’. All that needs to be done is to insert this missing ‘or any other person’, in this part of the definition as well. This small correction will ensure that those on inpatient treatment, along with their bystanders, would have the same set of rules applied, irrespective of whether they are in a government or a private-hospital-psychiatry-ward.

Yet another unacceptable provision in the Mental Health Act is the one whereby judicial officers can determine the presence and nature of mental illnesses in people, by personally ‘examining’ them. It is stated in Section 22[6] of the Act that the Magistrate ‘shall personally examine the alleged mentally ill person’! The same position is repeated in Sections 24 and 25 as well!

There is a widely prevalent misconception that unlike in other medical specialties, laymen could make psychiatric diagnosis! This is already causing a huge problem, in the entire mental health field! Priests, policemen and politicians all are supposed to have their expertise in Psychiatry! And now a Central Act requires judicial officers to ‘personally examine’ alleged patients! This kind of a ‘diagnosing’ of psychiatric illnesses by Magistrates would be a terrible blow to our Justice System!

The right position in Law ought to be that judicial officers must get persons suspected to be mentally ill, examined by qualified psychiatrists. And then on the basis of evidence deposed before them, by a psychiatrist, who has examined the concerned person, the Magistrate shall decide the issue. While the prerogative of giving judgment based on evidence and law belongs to Magistrates, determination of the presence of mental illness has to be done by qualified psychiatrists. This important issue has to be set right, by suitable modifications in the wording of Sections 22, 24 and 25 of the present Act.

In Chapter 1 on definitions, Section 2, Clause [l], says: ‘Mentally ill person means a person who is in need of treatment by reason of any mental disorder other than mental retardation’. Such an exclusion of mentally retarded persons from its preview is one more serious defect in the Act. The presumption here seems to be that ALL mentally retarded persons are being adequately taken care of, in institutions that are specially put up for them! This is a serious mistake. The fact is that only persons with ‘moderate’ and may be sometimes, even few victims of severe mental retardation, would be looked after in such institutions.

But what about those struck with profound mental retardation? They are often in states of acute excitement, impulsive violence and so on and their condition is often more pitiable than some of those ‘worst’ ‘mental’ patients![6] Facilities in homes for the retarded are not adequate to cope up with such very severely disturbed patients. They require expert treatment and care in psychiatric hospitals. But the present Law, by an explicit clause, denies them access to psychiatric hospitals, even in their state of utter helplessness!

The present legal impediment could be removed by deleting the words ‘excluding those with mental retardation’, in the definition of ‘psychiatric patients’ [Section 2, l]. It is also desirable to have a special provision in the new Act, to create some ‘Hospitals’, exclusively for the profoundly retarded. The Government has to assume responsibility in this area and provide the required kind of total guardianship to this extremely challenged section of people.

Some more ambiguities are there in the Section 2, on definitions. In clause [q], ‘psychiatric hospitals’ and ‘Psychiatric nursing homes’ are used as interchangeable. This is not an acceptable position. If two or more terms are to find a place in a ‘law-book’, each one of them should be given precise, distinct and clear definition.

In section [r] of the same Section 2, ‘Psychiatrist’ is defined. In the latter part of this definition, it is stated that ‘any medical officer who, having regard to his knowledge and experience in psychiatry, has been declared by the Government of that State to be a psychiatrist for the purpose of this Act’. This provision is obviously copied blindly from the old ‘Lunacy Act’,[9] which was drafted at a time when not too many psychiatrists were there. But for our current times, this is a totally unnecessary provision. It would only be misused by various Governments as was done by Government of Kerala, while posting an anesthesiologist as the ‘psychiatrist-member’ of the SMHA.

There are a few more instances where the wordings in the Act are causing some hitches in smooth patient-care. Chapter VI of the Act, which is a rather lengthy one, is dealing with ‘Judicial Inquisition’, regarding alleged mentally ill person possessing property. Terms like ‘lunatic’, ‘Lunacy Act’ and so on is used liberally in this chapter. This gives rise to an impression that those who prepared the draft-bill have copied the whole text from the old ‘Lunacy Act’, without a proper application of their minds! In the new Law this whole chapter has to be re-drafted. The objective is to bring in some brevity, as well as to ensure that this entire chapter has the required clarity.

While pointing out inadequacies, one should acknowledge that many good things are there in the Mental Health Act 1987. Take for example the provision of admission under special circumstances as per section 19 of the Act. This section, for admission AGAINST a patient's free-will, is indeed a great improvement from the old Lunacy Act days, when a reception order by a Court was mandatory, for all non-voluntary admissions! Today psychiatrists are legally empowered to order admission of unwilling patients, to mental hospitals.

But the question that each one of us should ask oneself is whether the profession is fully making use of Section 19, to meet difficult situations, for the good of patients? Or do we carry on with, what one may describe as, ‘forced voluntary admissions’ based on the good-old attitude that, ‘doctors know what is good for their patients, and where is the need for all these legal niceties?’

Another laudable provision is Section 23 [Chapter 4, Part B], where the powers and duties of police officers in respect to certain mentally ill persons have been laid dawn. If this provision is properly made use of, the plight of the mentally ill ‘living’ in conditions of total neglect and squalor, often in streets, could be considerably ameliorated. But enough has not been done so far, to ensure the indigent mentally ill benefit from Section 23 of the Act. Things could improve only when appropriate provisions are there in the Rules, which would force the Police to act positively, when a situation warrants.

Some more good things are there in the Act, like the special emphasis given to the human rights of patients, a provision to discharge court-ordered patients without having to wait for the court sanction and so on. While criticizing various defective provisions of the Act,[1] let us acknowledge with grace and gratitude, whatever is good and patient-friendly in the 1987 law. After all, our forefathers in the profession drafted it with an objective of securing a better deal for the mentally ill.[10,11]

Those who want to throw the present act lock-stock-and-barrel should not forget this. While we do our best to get all those provisions in the 1987-Act that are not good for psychiatry removed, all those patient-friendly provisions in the Law must be retained.[12,13] And by framing State Mental Health Rules with a clear-cut vision, we could certainly strive to give more teeth to the new Act.
